# Factors Driving Inequality in Prostate Cancer Survival: A Population Based Study

**DOI:** 10.1371/journal.pone.0106456

**Published:** 2014-09-09

**Authors:** Richéal M. Burns, Linda Sharp, Francis J. Sullivan, Sandra E. Deady, Frances J. Drummond, Ciaran O′Neill

**Affiliations:** 1 Health Economic Research Centre, Nuffield Department of Population Health, University of Oxford, Oxford, United Kingdom; 2 Surgical Intervention Trials Unit, Nuffield Department of Surgical Science, University of Oxford, Oxford, United Kingdom; 3 National Cancer Registry, Cork, Ireland; 4 Prostate Cancer Institute, NUI Galway, Galway, Ireland; 5 J.E. Cairnes School of Business and Economics, NUI Galway, Galway, Ireland; Baylor College of Medicine, United States of America

## Abstract

**Purpose:**

As cancer control strategies have become more successful, issues around survival have become increasingly important to researchers and policy makers. The aim of this study was to examine the role of a range of clinical and socio-demographic variables in explaining variations in survival after a prostate cancer diagnosis, paying particular attention to the role of healthcare provider(s) i.e. private versus public status.

**Methods:**

Data were extracted from the National Cancer Registry Ireland, for patients diagnosed with prostate cancer from 1998–2009 (N = 26,183). A series of multivariate Cox and logistic regression models were used to examine the role of healthcare provider and socio-economic status (area-based deprivation) on survival, controlling for age, stage, Gleason grade, marital status and region of residence. Survival was based on all-cause mortality.

**Results:**

Older individuals who were treated in a private care setting were more likely to have survived than those who had not, when other factors were controlled for. Differences were evident with respect to marital status, region of residence, clinical stage and Gleason grade. The effect of socio-economic status was modified by healthcare provider, such that risk of death was higher in those men of lower socio-economic status treated by public, but not private providers in the Cox models. The logistic models revealed a socio-economic gradient in risk of death overall; the gradient was larger for those treated by public providers compared to those treated by private providers when controlling for a range of other confounding factors.

**Conclusion:**

The role of healthcare provider and socio-economic status in survival of men with prostate cancer may give rise to concerns that warrant further investigation.

## Introduction

As cancer control strategies become more successful, issues around survivorship have begun to receive more attention from researchers and policy makers. Identifying variations in survivorship and seeking to explain these among cancer patients has attracted particular attention [1]–[3]. The incidence and survival rates in prostate cancer offers a greater opportunity to examine variations in survival compared with other cancers. Prostate cancer is now the most commonly diagnosed cancer in men in developed countries [4]. Survival prospects for men are good: the mean European age and area-standardised 5-year survival for men diagnosed in 1995–99 was 76% and the 5-year relative survival in the Republic of Ireland (RoI) was 88% for patients diagnosed in 2004-07 [5]–[6]. In the USA, prostate cancer survivors comprise an estimated 43% of male cancer survivors [7] and large numbers of men survive over relatively long periods of time [8].

In the RoI, approximately 2,500 men are diagnosed annually with prostate cancer [9]. The RoI was estimated to have the highest incidence of prostate cancer across Europe in 2006 and 2008 and the fifth highest prostate cancer mortality rate in Europe [10]–[12]. Previous studies have highlighted the role of non-need factors, namely voluntary private health insurance, in the uptake of PSA testing in the RoI and across Europe [13]–[14]. Within this context the examination of survival with respect to prostate cancer in Ireland is opportune. The aim of this study was to examine what role, if any, location of care i.e. public versus private, for prostate cancer had on all-cause survival, controlling for a range of clinical and socio-demographic variables.

## Materials and Methods

### Study setting

The RoI has a mixed public-private healthcare system. All citizens are entitled to the standard level of care within the public system with some co-payments (including prescription charges and General Practitioner (GP) charges for those above a given level of income). Approximately one third of the population are entitled to free health care services and medicines under the Health Service Executive (HSE) General Medical Services (GMS) scheme based on income and/or age criteria [15]. During the period of this study, approximately half of the population held private health insurance (PHI), with insurance plans which mainly cover the costs of in-patient stays and outpatient visits; PHI is perceived by many to provide speedier access to care [16]. There are 51 public hospitals in the country, including 8 designated cancer centres; however, cancer patients are also treated outside these cancer centres [17]. Many of the public hospitals contain some private beds, which patients with PHI may choose to use. There are also a growing number of private hospitals (n = 24), some of which undertake cancer surgery and/or provide radiotherapy [17].

Approximately 83% of total acute public hospital discharges (N = 1,332,680 excluding maternity) in 2011 were categorised as being treated on a public basis [18]. In 2004, the government set up a statutory body, the National Treatment Purchase Fund (NTPF), to alleviate the long waiting lists for public patients; the NTPF monitors public patients and purchases treatment for those who have been on waiting lists for over three months from private hospitals [19]. The proportion of public patients treated in a private setting under this initiative is small; in 2010 the total in-patient services carried out under the NTPF was 24,118 (<2% of overall total discharges), with urology and radiology accounting for on average 11% of all NTPF procedures [20]. Therefore, the majority of patients treated in a public setting do not obtain private health care, suggesting they do not hold private insurance, and the majority of patients treated in a private setting appear to do so based on the possession of PHI.

### Data

The NCR has permission under the Health (Provision of Information) Act 1997 to collect and hold data on all persons diagnosed with cancer in Ireland. The use of that data for research is covered by the Statutory Instrument which established the Registry Board in 1991. All datasets were anonymised prior to analysis. Data were extracted from the National Cancer Registry Ireland (NCRI), for all patients diagnosed with prostate cancer (ICD10 C61) during 1998–2009 inclusive (N = 26,938). Men with incomplete records in relation to age (n = 18), clinical staging (n = 8), county of residence (n = 9) and those diagnosed through autopsy (n = 87), were excluded (total n = 122). The remaining patients (n = 26,816) were stratified into three age-groups, 35 to 54 years, 55 to 69 years and ≥70 years, based on those used in the European Randomised Study of Screening for Prostate Cancer (ERSPC) and previous analyses [13]–[14], [21]. Five socio-economic groups were constructed using a national, standardised, area-based measure of deprivation for patients' place of residence at diagnosis with ‘SES1’ being the highest and ‘SES5’ the lowest socio-economic grouping [22]–[23]. A sixth group, ‘SESunk’ was constructed for those who were unclassified i.e. patients whose addresses were insufficiently precise to be able to allocate them to a deprivation category (n = 2,771). Cases were also classified according to the province of residence (of which there are four in the RoI). Patients were classified according to whether or not they were married or living as such at diagnosis and a binary variable for smoking status was constructed reflecting whether or not the man was a smoker at the time of diagnosis based on medical record information recorded at the time of registration.

In terms of clinical variables, cases were grouped according to the way in which they presented i.e. opportunistic “screening,” incidental (discovery during the course of another investigation or treatment including Transurethral Resection of the Prostate (TURP)), symptomatic, and ‘other’. Data was available on Gleason grade and clinical and pathological classification TNM. Gleason scores range from 2 to 10 and four categories were constructed: grade 1, Gleason grade <5; grade 2, Gleason grade 5–7; grade 3, Gleason grade >7 and grade 4- undifferentiated tumour grade; a binary variable for grade unknown was also constructed [24]. In keeping with clinical guidelines, each prostate cancer was characterised in terms of summary stage, in five categories (stage I–IV, and unknown) [25]. Additional analyses revealed that the majority of patients with unknown grade and stage were over 70 years and received treatment similar to those with late stage prostate cancer (data not shown); therefore assuming a hierarchical ordering, this suggests stage unknown is similar to stage 4 and grade unknown is similar to grade 4; individual survival estimates supports this assumption.

A variable classifying patients according to the likelihood of public/private status of their prostate cancer healthcare provider(s) was constructed as follows. Each hospital at which a patient is seen in the first year following diagnosis is recorded by the NCRI. Since it was not possible to identify the type of bed (i.e. public or private), occupied by a patient in a public hospital, for this analysis “public” patients were defined as those who attended only public hospitals during the first year of their treatment. In the same manner, those who used only private facilities for their treatment were classified as “private” patients. A third group, those who received part of their care privately was also constructed. These categories can be thought of as representing the observed counterpart of the unobserved variable i.e. likelihood of being a private patient. Patients treated publicly only being likely to be public patients, those treated partly in the public and partly in the private system being less likely to be public patients and those treated solely in the private sector being unlikely to be public patients.

Deaths in those diagnosed with cancer are identified by the NCRI by routine linkage with death certificates. For this study, information on deaths was complete until 31/12/2010 (thus, all patients had at least one year follow-up). Deaths from all-causes were considered; information on cause-of-death is not generally publicly available at the level of the individual. All data used in our analyses are available from the National Cancer Registry Ireland with standard terms and conditions for data release, use and reporting. Further information about this is available from the Registry website (http://www.ncri.ie/content/conditions-use-national-cancer-registry-data).

### Analysis

Survival time was calculated in months, from date of diagnosis, with censoring applied for varying follow-up periods: (i) the entire follow-up period available (i.e. to 31/12/2010); (ii) 3-year (36 months) follow-up; and (iii) 7-year (84 months) follow-up.

The impact of socio-economic status and healthcare provider(s) i.e. private versus public, on survival was examined in three ways; the three approaches were used for confirmatory purposes as well as for purposes of exposition. Firstly, a series of Cox proportional hazards models were run exploring the role of healthcare provider alone (i.e. unadjusted for socio-demographic and clinical confounders) by year of diagnosis. The assumptions, strengths and weaknesses of employing a Cox proportional hazards regression model for survival analysis in cancer have been extensively discussed elsewhere [26]–[27]. One limitation of the conventional Cox model is that the results are valid and interpretable only when hazards are proportional over time. Non-proportional hazards were evident in this data with respect to socio-economic status in particular. Thus in our second approach, a series of stratified, Cox non-proportional hazards models were run examining socio-economic status controlling for a range of covariates including healthcare provider, using a conditional approach [28]. Categorical variables for socio-economic status were tested for joint significance using Wald tests and Global tests were employed for each Cox regression model to measure model appropriateness. Thirdly, a series of logistic models were undertaken; in these models the outcome of interest was a binary variable for vital status – alive or dead. These models assessed the impact of a wider range of demographic and clinical explanatory variables, including interactions, on risk of death; thus providing impacts of a wider range of contributory factors on survival in a confirmatory manner albeit not addressing time dependency on covariates. Wald tests for clinical variables (i.e. grade and stage) and for demographics (e.g. region of residence) and socio-economic status were performed, while models were clustered by year of incidence and logistic post estimation techniques including classification competency and the Hosmer-Lemeshow goodness of fit statistic were also calculated [29].

## Results

26,816 men diagnosed with prostate cancer from 1998 to 2009 were included in the analysis. For the analysis based on 3-years follow-up, 79% of men (16,116 of the 20,507 men diagnosed in 1998–2007) survived for 36 months or longer. For the analysis based on 7 years follow-up, 55% of men (5,634 of the 10,310 diagnosed from 1998–2003) survived for 84 months or longer. *Table 1* details descriptive statistics for the entire study population. Those treated solely in a public healthcare setting accounted for 70% (n = 18,683) of those diagnosed and the remaining 30% (n = 8,133) were classified as receiving private care in part or wholly during their treatment pathway: 17% (n = 4,465) were treated solely in a private setting and 13% (n = 3,668) received care in both public and private settings.

**Table 1:** Characteristics of prostate cancer cases diagnosed from 1998–2009^1^, included in analysis.

Variable Name	Number of observations	Variable Name	Number of observations
Health provider: Public	18683 (70%)	Marital status: Married	17715 (66%)
Private/Mixed	8133 (30%)	Single/Divorced/Widowed/Unknown	9101 (34%)
Socio-economic status: SES1	4756 (18%)	MOP^2^: Other	6338 (24%)
SES2	3138 (12%)	Screen- opportunistic	4105 (15%)
SES3	3657 (14%)	Incidental	2854 (11%)
SES4	4708 (18%)	Symptomatic	13519 (50%)
SES5	7786 (29%)	Smoker at diagnosis: No	23129 (86%)
SES Unk	2771 (9%)	Yes	3687 (14%)
Stage: Stage I	872 (3%)	Province: Leinster	12434 (46%)
Stage II	14009 (52%)	Ulster	7958 (30%)
Stage III	2252 (8%)	Connacht	4406 (16%)
Stage IV	3111 (12%)	Munster	2018 (8%)
Stage Unknown	6572 (25%)	Age: 35–54 years	1579 (6%)
Grade: Gleason <5	1932 (7%)	55–69 years	12224 (45%)
Gleason 5–7	14324 (53%)	70 years and over	13013 (49%)
Gleason >7	5131 (19%)		
Undifferentiated	85 (<1%)		
Grade Unknown	5344 (20%)		

1.) 26,938 diagnosed in 1998–2009 but (n = 122) excluded from analysis due to missing data.

2.) MOP- method of presentation.

3.) SES Unk- Socio-economic status unknown.

**Figure 1:**
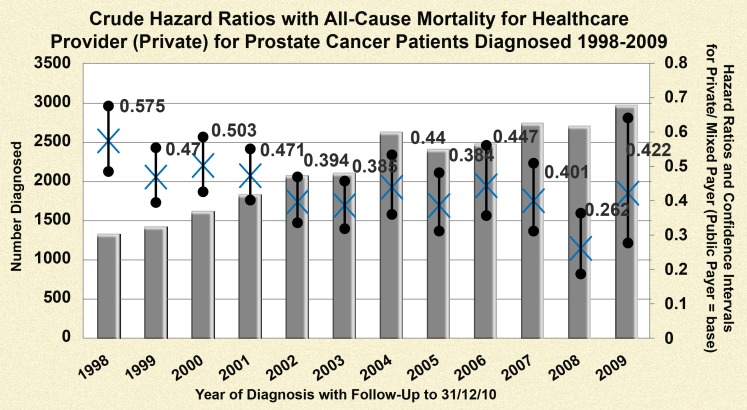
Crude hazard ratios for healthcare provider by year of diagnosis for men diagnosed with prostate cancer (1998–2009). **a**. This graph contains 12 individual Cox PH models where the base category is ‘Public Payer’ for varying follow-up periods up to 31/12/10. **b**. All Hazard Ratios presented are statistically significant and confidence intervals are depicted by the line segments. **c** The test of proportional hazards (global test) revealed marginal non-proportionality for models with the following year of diagnosis: 1998, 2002, 2003, 2004; therefore caution is warranted in interpretation; however overall men with access to private healthcare had a lower risk of death than those who did not have access.

Crude hazard ratios for a series of Cox proportional hazard regression models examining the impact of healthcare provider on all-cause mortality by year of diagnosis are illustrated in *Figure 1*; these are shown only for the entire follow-up period available (i.e. to 31/12/2010). The hazard ratio fluctuated by year of diagnosis and on average, those who were seen or treated at any time in the first year post-diagnosis in a private healthcare setting had a statistically significant reduced risk of death compared to those seen or treated solely in a public setting (univariate HR 0.43 (95% CI: 0.41, 0.45)).

When controlled for age and clinical factors i.e. stage and grade, the effect of healthcare provider was diminished but remained statistically significant for the entire follow-up period (HR: 0.608; 95% CI: 0.573, 0.644). Over a 36 month follow-up a 42% reduced hazard of death was observed (HR: 0.577; 95% CI: 0.530, 0.628), and a 37% reduced hazard was observed at 84 months (HR: 0.628; CI: 0.581, 0.679).

Results from the stratified, interaction Cox regression models for non-proportional hazards are presented on *Tables 2* and *3*. Over both follow-up times, a significant role accorded to socio-economic status was evidenced when stratified by non-need factors i.e. healthcare provider, marital status and region of residence. Patients treated in a public setting from the lowest socio-economic group had a 21% (P<0.01) increased hazard of death over the 36 month follow-up and a 25% (P<0.01) increased hazard of death over the 84 month follow-up compared to those from the highest socio-economic group. However, patients treated in part or wholly in a private setting exhibited no social gradient for either follow-up time in the non-proportional hazard models.

**Table 2:** Multivariate stratified Cox regression for 36 month survival.

Stratified interaction non-PH Cox regression Model (36 month follow-up)				
	Private	Public	Married	Not Married
SES1	1	1	1	1
SES2	1.02	1.19***	1.34***	1.09
SES3	0.95	1.01	1.10	1.03
SES4	1.00	1.16**	1.31***	1.08
SES5	1.05	1.21***	1.43***	1.15**
SES Unknown	0.85	1.15**	1.14	1.15
Global Test	8.22 (P = 0.14)	4.88 (P = 0.43)	7.09 (P = 0.21)	8.88 (P = 0.11)
Wald Test *SES group	Chi2(5) = 2.02 (P = 0.85)	Chi2(5) = 21.31 (P = 0.00)	Chi2(5) = 42.67 (P = 0.00)	Chi2(5) = 5.54 (P = 0.35)
Number of Observations	6191	14316	13929	6578
	Gleason grade 5–7	Gleason grade >7	Stage II	Stage III
SES1	1	1	1	1
SES2	1.46***	1.16	1.58***	1.23
SES3	1.20**	1.05	1.24*	0.94
SES4	1.29**	1.20**	1.40***	1.02
SES5	1.58***	1.24***	1.51***	1.46*
SES Unknown	1.36***	1.19	1.35**	0.98
Global Test	6.98 (P = 0.22)	4.36 (P = 0.50)	12.03 (P = 0.04)	6.07 (P = 0.29)
Wald Test *SES group	Chi2(5) = 29.24 (P = 0.00)	Chi2(5) = 9.01 (P = 0.10)	Chi2(5) = 23.50 (P = 0.00)	Chi2(5) = 6.08 (P = 0.30)
Number of Observations	11105	3881	10306	1690
	Leinster	Connacht	Munster	Ulster
SES1	1	1	1	1
SES2	1.41***	1.21	1.00	0.75
SES3	1.16*	0.90	1.00	0.95
SES4	1.29***	1.27*	1.00	1.03
SES5	1.59***	1.17	1.17*	0.79
SES Unknown	1.30***	1.10	1.03	0.60
Global Test	5.18 (P = 0.39)	9.89 (P = 0.08)	4.09 (P = 0.54)	3.88 (P = 0.57)
Wald Test *SES group	Chi2(5) = 55.04 (P = 0.00)	Chi2(5) = 9.39 (P = 0.09)	Chi2(5) = 7.50 (P = 0.19)	Chi2(5) = 7.03 (P = 0.22)
Number of Observations	9598	3250	6125	1534

Notes: 1.) Hazard Ratios for not surviving at 36 months reported with clustered standard errors.

2.) Significance: * (P<0.10), ** (P<0.05), *** (P<0.01).

3.) “SES”- Socio-economic status.

**Table 3:** Multivariate stratified Cox regression for 84 month survival.

Stratified interaction non-PH Cox regression Model (84 month follow-up)				
	Private	Public	Married	Not Married
SES1	1	1	1	1
SES2	1.15	1.24***	1.34***	1.19*
SES3	0.94	1.07	1.12*	1.11
SES4	0.97	1.18***	1.23***	1.17**
SES5	1.09	1.25***	1.39***	1.22***
SES Unknown	0.81	1.21***	1.16*	1.16
Global Test	2.22 (P = 0.82)	3.26 (P = 0.66)	6.29 (P = 0.28)	6.72 (P = 0.24)
Wald Test *SES group	Chi2(5) = 6.73 (P = 0.24)	Chi2(5) = 24.17 (P = 0.00)	Chi2(5) = 38.83 (P = 0.00)	Chi2(5) = 8.36 (P = 0.13)
Number of Observations	2902	7408	6875	3435
	Gleason grade 5–7	Gleason grade >7	Stage II	Stage III
SES1	1	1	1	1
SES2	1.38***	1.25**	1.48***	1.64*
SES3	1.21**	1.05	1.28**	1.58*
SES4	1.19**	1.28***	1.27**	1.62**
SES5	1.58***	1.30***	1.50***	1.83***
SES Unknown	1.24*	1.15	1.24*	0.93
Global Test	4.29 (P = 0.49)	2.13 (P = 0.83)	4.56 (P = 0.47)	4.94 (P = 0.42)
Wald Test *SES group	Chi2(5) = 38.67 (P = 0.00)	Chi2(5) = 13.92 (P = 0.02)	Chi2(5) = 24.84 (P = 0.00)	Chi2(5) = 10.43 (P = 0.06)
Number of Observations	4753	2041	4119	748
	Leinster	Connacht	Munster	Ulster
SES1	1	1	1	1
SES2	1.35***	1.47**	1.06	0.80
SES3	1.15*	1.23	0.93	0.97
SES4	1.26***	1.35**	1.03	0.97
SES5	1.59***	1.31*	1.10	0.86
SES Unknown	1.28**	1.18	1.08	0.59
Global Test	0.78 (P = 0.97)	4.47 (P = 0.48)	2.45 (0.78)	5.59 (P = 0.35)
Wald Test *SES group	Chi2(5) = 61.31 P = 0.00)	Chi2(5) = 7.96 (P = 0.15)	Chi2(5) = 4.81 (P = 0.44)	Chi2(5) = 4.24 (P = 0.51)
Number of Observations	4901	1458	3118	833

Notes: 1.) Hazard Ratios for not surviving at 36 months reported with clustered standard errors.

2.) Significance: * (P<0.10), ** (P<0.05), *** (P<0.01).

3.) “SES”- Socio-economic status.

A social gradient in risk of death was also apparent for the Gleason grade (5–7, >7) and clinical stage (II, III) strata; those from lower socio-economic groups had a higher risk of mortality across both follow-up time periods. Patients from the lowest socio-economic group (SES5) with Gleason grade 5–7, and >7 had a 58% (P<0.01) and a 30% (P<0.01) increased risk of death, respectively compared to the highest socio-economic group, over the 84 month follow-up period. The same patterns were seen in the analysis of the entire follow-up available (data not shown).

Results of the logistic regression analyses of risk of death are reported in *Table 4*, for the three periods of follow-up (all, 36 months, 84 months). In the logistic models, healthcare provider was not statistically significant when a wider range of covariates were controlled for. However, there was a significant interaction between healthcare provider and age: those aged 70 and over (representing approximately 50% of the study cohort) treated in part or wholly in a private setting were 46% (P<0.01) overall, 49% (P<0.01) at 36 months post-diagnosis and 52% (P<0.01) at 84 months post-diagnosis less likely to die compared to the those under 70 years of age and treated solely in a public setting over the respective follow-up periods. The logistic analyses also highlighted a social gradient with patients from the lowest socio-economic group being 26% (P<0.01) more likely to die at 36 months post-diagnosis and 34% (P<0.01) more likely to die at 84 months post-diagnosis compared to the highest socio-economic group; thus confirming the presence of socio-economic inequality when a wider range of confounding factors were controlled for. When interaction terms for socio-economic status and healthcare provider (private) were also included in logistic models (results not shown), no statistically significant impact on mortality was evident for this term. That an effect was detected in the Cox but not logistic models may reflect the greater sensitivity of a model using time to event rather than event within a defined time in this particular case. A further set of logistic analyses was undertaken stratifying by healthcare status highlighted in *Table 5*; in the 84 month post-diagnosis follow-up, those with access to private care from the lowest socio-economic group were 28% (P<0.01) more likely to die than those from the highest socio-economic group; among those with access to public healthcare only, those from the lowest socio-economic group were 33% (P<0.01) more likely to die than those from the highest socio-economic group.

**Table 4:** Multivariate logistic regression analysis (odds ratios reported).

Logistic analyses at 36 months & 84 months follow-up			
Dependant: Deceased at date of censoring = 1 and 0 otherwise	Overall	36+ months	84+ months
Health provider: Public	1	1	1
Private/Mixed	0.94 (CI:0.64, 1.36)	0.93 (CI:0.64, 1.36)	1.04 (CI: 0.57, 1.90)
Age: 35–54 years	1	1	1
55–69 years	2.06*** (CI:1.62, 2.62)	2.01*** (CI: 1.53, 2.64)	2.08*** (CI:1.34, 3.25)
70 and over years	9.08*** (CI:7.01, 11.74)	9.23*** (CI: 6.86, 12.42)	11.26*** (CI:7.05, 17.96)
Interactions: 55–69 years/Private	0.77 (CI: 0.55, 1.10)	0.73* (CI:0.51, 1.05)	0.66 (CI:0.37, 1.17)
70 years and over/Private	0.54*** (CI:0.39, 0.76)	0.51*** (CI:0.37, 0.72)	0.48*** (CI:0.28, 0.82)
Socio-economic status: SES1	1	1	1
SES2	1.17*** (CI:1.05, 1.30)	1.22*** (CI:1.08, 1.37)	1.33*** (CI:1.15, 1.53)
SES3	1.12*** (CI:1.02, 1.24)	1.14** (CI:1.02, 1.27)	1.22*** (CI:1.08, 1.37)
SES4	1.08 (CI:0.97, 1.19)	1.10 (CI:0.97, 1.24)	1.19** (1.02, 1.39)
SES5	1.20*** (CI:1.09, 1.33)	1.26*** (CI:1.14, 1.39)	1.34*** (CI:1.18, 1.52)
SES Unknown	0.89 (CI:0.76, 1.05)	1.02 (CI:0.87, 1.19)	1.24** (CI:1.01, 1.53)
Stage: Stage I	1	1	1
Stage II	1.21 (CI:0.94, 1.55)	1.23 (CI:0.95, 1.59)	1.13 (CI:0.81, 1.58)
Stage III	1.28 (CI:0.93, 1.78)	1.35* (CI:0.98, 1.85)	1.30 (CI:0.93, 1.82)
Stage IV	9.37*** (CI:7.16, 12.26)	9.83*** (CI:7.61, 12.70)	7.94*** (CI:6.30, 10.02)
Stage Unknown	3.21*** (CI:2.42, 4.27)	2.88*** (CI:2.13, 3.88)	1.95*** (CI:1.56, 2.43)
Grade: Gleason <5	1	1	1
Gleason 5–7	0.70*** (0.60, 0.83)	0.73*** (CI:0.62, 0.86)	0.87* (CI:0.76, 1.01)
Gleason >7	1.48** (CI:1.08, 2.04)	1.85*** (CI:1.50, 2.29)	2.47*** (CI:2.06, 2.94)
Grade Undifferentiated	2.13*** (CI:1.20, 3.76)	2.87*** (CI:1.46, 5.65)	3.50** (CI:1.03, 11.90)
Gleason grade Unknown	1.96*** (CI:1.53, 2.50)	2.10*** (1.70, 2.60)	2.21*** (CI:2.00, 2.44)
Marital Status: Single/Divorced/Widowed	1	1	1
Married	0.80*** (CI:0.71, 0.91)	0.75*** (CI:0.68, 0.82)	0.71*** (CI:0.64, 0.79)
Region: Leinster	1	1	1
Ulster	0.55*** (CI:0.45, 0.66)	0.54*** (0.43, 0.69)	0.56*** (0.43, 0.73)
Connacht	0.83*** (CI:0.72, 0.96)	0.87** (0.76, 0.99)	0.87 (0.68, 1.13)
Munster	1.31*** (CI:1.20, 1.42)	1.30*** (1.18, 1.43)	1.16*** (1.08, 1.24)
MOP: Other	1	1	1
Screen- opportunistic	0.39*** (0.26, 0.58)	0.37*** (CI:0.25, 0.57)	0.47*** (CI:0.29, 0.77)
Incidental	1.08 (CI:0.85, 1.38)	0.95 (CI:0.76, 1.18)	0.83*** (CI:0.74, 0.93)
Symptomatic	1.59*** (CI:1.17, 2.17)	1.37*** (CI:1.13, 1.67)	1.28*** (CI:1.18, 1.40)
Smoker at diagnosis: No	1	1	1
Yes	1.73*** (CI:1.59, 1.89)	1.64*** (CI:1.52, 1.77)	1.63*** (CI:1.43, 1.86)
Number of Obs	26816	21054	10697
Wald test for Stage and Gleason Grade	Chi2 (8) = 1640 (P = 0.00)	Chi2 (8) = 41488 (P = 0.00)	Chi2 (5) = 1427 (P = 0.00)
Wald test for SES & Region	Chi2 (8) = 850 (P = 0.00)	Chi2 (8) = 3162 (P = 0.00)	Chi2 (5) = 93.91 (P = 0.00)
% Correctly Classified	79.80%	78.22%	76.37%

Notes: 1.) Odds Ratios with clustered standard errors (Confidence Intervals in brackets).

2.) Significance: * (P<0.10), ** (P<0.05), *** (P<0.01).

3.) “SES”- Socio-economic status.

4.) Interactions between socio-economic status and healthcare provider were also included in the logistic analyses but not reported above due to lack of statistical significance across all models.

**Table 5:** Multivariate logistic regression analysis stratified by healthcare payer (odds ratios reported).

Stratified logistic analysis at 84 months follow-up		
Dependant: Deceased at date of censoring = 1 and 0 otherwise	Private = 1	Private = 0
Age: 35–54 years	1	1
55–69 years	1.37 (CI:0.68, 2.79)	2.05*** (CI:1.33, 3.15)
70 and over years	5.76*** (CI:3.47, 9.57)	10.82*** (CI:6.88, 17.00)
Socio-economic status: SES1	1	1
SES2	1.21 (CI:0.88, 1.66)	1.31*** (CI:1.14, 1.50)
SES3	1.07 (CI:0.77, 1.50)	1.21*** (CI:1.07, 1.37)
SES4	1.22*** (CI:1.07, 1.39)	1.18** (1.01, 1.37)
SES5	1.28*** (CI:1.04, 1.57)	1.33*** (CI:1.15, 1.52)
SES Unknown	1.04 (CI:0.80, 1.35)	1.21* (CI:0.97, 1.52)
Stage: Stage I	1	1
Stage II	0.58** (CI:0.36, 0.92)	1.34 (CI:0.93, 1.96)
Stage III	0.59** (CI:0.37, 0.93)	1.64*** (CI:1.16, 2.31)
Stage IV	5.30*** (CI:3.97, 7.08)	8.67*** (CI:6.42, 11.72)
Stage Unknown	0.89 (CI:0.67, 1.19)	2.42*** (CI:1.85, 3.18)
Grade: Gleason <5	1	1
Gleason 5–7	0.99 (0.83, 1.17)	0.84* (CI:0.68, 1.03)
Gleason >7	3.45*** (CI:2.10, 5.65)	2.07*** (CI:1.64, 2.59)
Grade Undifferentiated	12.82 (CI:0.57, 285.86)	2.68* (CI:0.86, 8.33)
Gleason grade Unknown	1.46*** (CI:1.08, 1.98)	2.39*** (CI:2.17, 2.63)
Marital Status: Single/Divorced/Widowed	1	1
Married	0.83** (CI:0.69, 1.00)	0.68*** (CI:0.62, 0.75)
Region: Leinster	1	1
Ulster	0.61*** (CI:0.46, 0.83)	0.54*** (0.40, 0.73)
Connacht	0.77 (CI:0.46, 1.27)	0.93 (0.72, 1.20)
Munster	0.90 (CI:0.70, 1.17)	1.22** (1.02, 1.46)
MOP: Other	1	1
Screen- opportunistic	0.77 (0.53, 1.13)	0.42*** (CI:0.24, 0.74)
Incidental	0.86 (CI:0.65, 1.14)	0.84*** (CI:0.74, 0.95)
Symptomatic	1.36*** (CI:1.18, 2.10)	1.29*** (CI:1.20, 1.40)
Smoker at diagnosis: No	1	1
Yes	1.58*** (CI:1.19, 1.56)	1.65*** (CI:1.45, 1.87)
Number of Obs	2917	7780
Wald test for Stage and Gleason Grade	Chi2 (5) = 128 (P = 0.00)	Chi2 (5) = 868 (P = 0.00)
Wald test for SES & Region	Chi2 (5) = 11 (P = 0.05)	Chi2 (5) = 78 (P = 0.00)
% Correctly Classified	74.87%	77.07%

Notes: 1.) Odds Ratios with clustered standard errors (Confidence Intervals in brackets).

2.) Significance: * (P<0.10), ** (P<0.05), *** (P<0.01).

3.) “SES”- Socio-economic status.

## Discussion

Variations in survival after a diagnosis of cancer have attracted increased attention by researchers. The analyses carried out here highlights that patients treated in a private healthcare setting had an average of 40% reduced risk of mortality compared to those who were treated solely in the public setting, when adjusted for age and clinical variables. It is also evident from these results that, after controlling for stage, grade, marital status, healthcare setting and region of residence, there was a clear socio-economic gradient in survival. Moreover, socio-economic status and healthcare provider interacted to influence risk of mortality in proportional hazard models. Patients who accessed public healthcare provision from the lowest socio-economic group had approximately 21–25% increased risk of death compared to those from the highest socio-economic group; this gradient was not evident for patients who were seen by a private provider when addressing time dependency. Care, however, is warranted in the interpretation of this result.

A number of studies have examined the relationship between health insurance status and cancer service utilization in Ireland and internationally [13], [30]–[32]. The evidence here relates to where patients were treated rather than insurance status directly and is open to different interpretations. While it is possible that there exist differences in the quality of care provided in the public and private systems that directly impacts upon survival, it must be remembered that the mortality examined here is all-cause mortality. Consequently it is also possible that the differences in survival observed between patients treated in public and private healthcare settings relate more to differences in patient characteristics than the care received in respect of prostate cancer.

While we adjusted for a range of clinical and socio-demographic factors, we were not able to adjust for many other factors that may differ between public and privately-treated patients and that may affect survival. These could include, for example, PSA level at diagnosis, patient preferences, functional and health status, and various other health-related and non-health-related (e.g. availability of social support) indicators of suitability for curative treatment. Moreover, selection effects may exist in terms of lifestyle between those who consume care in private and public facilities; the former may have unobserved healthier behaviours that can explain differences in survival, independent of the care received. As noted the need for caution here is acute given the use of all-cause mortality in this study; those dying with prostate cancer rather than from prostate cancer may exhibit unique clinical and environmental characteristics that could not be controlled for in this analysis [6].

This analysis had several limitations. Firstly, the analysis examined all-cause mortality due to lack of availability of patient-level information on cause of death; as stated above caution is necessary in interpretation, however previous analyses have found marginal survival differences in all-cause and excess mortality in prostate cancer [33]. In addition, data were not available on co-morbidities and therefore this could not be controlled for; those from lower socio-economic groups traditionally have higher co-morbidities than those from higher socio-economic groups which may partly explain the social gradient evidenced [34]. However, a recent study investigating confounding factors in curative treatments for prostate cancer patients in the RoI found no significant role accorded to co-morbidities [35]. The categorical variables for stage, grade and socio-economic status all contained an ‘unknown’ category which was included in the analysis for purposes of completeness. It was assumed based on cross-tabulations with both clinical and non-need factors that each of these ‘unknown’ categories exhibited a moderate ordering with respect to their defined counterparts. This assumption was given more weight when output from the various regression models was examined. Finally, as discussed, the available data did not permit breakdown of those cared for within the public system by whether they were seen as private or public patients.

As increasing numbers of patients survive cancer, interest in differences in survival patterns will increase. Given the high incidence of prostate cancer in the RoI and internationally, a better understanding of the determinants of survival will provide policy makers and healthcare professionals with much needed evidence to improve both access to and delivery of care. In this analysis, a socio-economic gradient was evident, but the magnitude of the effect varied considerably dependent upon the sub groups analysed as well as the follow-up time periods. The results with respect to healthcare provision may give rise to concerns but care is warranted in the interpretation and further analysis of them required to establish whether concerns are legitimate or misplaced.
